# Supplementary material for: Leaving academia: PhD attrition and unhealthy research environments
Source: PLoS One. 2022 Oct 5;17(10):e0274976. doi: 10.1371/journal.pone.0274976 (PMC9534392; doi:10.1371/journal.pone.0274976)
Supplement: S1 File — (DOCX) [file pone.0274976.s001.docx]

All supporting information can be found in the OSF repository of the project: <https://osf.io/bqx7v/>
