# Supplementary material for: Leaving academia: PhD attrition and unhealthy research environments
Source: PLoS One. 2022 Oct 5;17(10):e0274976. doi: 10.1371/journal.pone.0274976 (PMC9534392; doi:10.1371/journal.pone.0274976)
Supplement: Attachment — Submitted filename: PLOS One review.docx [file pone.0274976.s002.docx]

**PLOS One review: Leaving academia**

This is an interesting and unique study of PhD candidates’ views of leaving academia. I have some suggestions for additions, changes, and clarifications:

- Mention the research methods used in the abstract and introduction.
- In the introduction, briefly acknowledge reasons for leaving other than those explored specifically in the study (e.g., lack of career prospects).
- Some of the sections overlap somewhat: e.g., 1. and 2.1 has some repetition; 3.1 describes context rather than methods.
- Express percentages consistently (e.g., with single, double, or no decimal points).
- Include footnote 1 in the main text, as it’s important to some of your later discussion.

There are also a few areas in the paper where the authors make assumptions that have been explored (and challenged) through previous research on leaving academia. For instance, regarding the desirability of ‘passion’ for academic research, which can negatively impact scholars, leading them to accept long-term precarious roles.

There is also the question of the desirability of low attrition (which is often presumed in studies of doctoral attrition, as well as by university managers, governments, and funding bodies). However, such scholarship ignores the fact that PhD programs have become more and more time limited (and poorly funded) in recent years. It also tends to avoid questions about the difficulty of PhD studies, and the role that research methods and resourcing play in influencing time to completion (e.g., the time commitment of intensive qualitative research, or the impact of being in a poorly funded discipline where supervisors are overworked).

Some research to cite includes:

- Barcan, R. 2019. Weighing up futures: experiences of giving up an academic career, in C. Manathunga and D. Bottrell (eds.), Resisting neoliberalism in higher education volume II: prising open the cracks, 43–64. Basingstoke: Palgrave Macmillan.
- Coin, F. 2017. ‘On quitting: the labour of academia’, Ephemera: Theory and Politics in Organization 17: 705–19.
- McKenzie, Lara 2021. ‘Unequal expressions: emotions and narratives of leaving and remaining in precarious academia’, Social Anthropology 29(2): 527–542.
